# Expert-guided optimization for 3D printing of soft and liquid materials

**DOI:** 10.1371/journal.pone.0194890

**Published:** 2018-04-05

**Authors:** Sara Abdollahi, Alexander Davis, John H. Miller, Adam W. Feinberg

**Affiliations:** 1 Department of Biomedical Engineering, Carnegie Mellon University, Pittsburgh, Pennsylvania, United States of America; 2 Department of Engineering & Public Policy, Carnegie Mellon University, Pittsburgh, Pennsylvania, United States of America; 3 Department of Social and Decision Sciences, Carnegie Mellon University, Pittsburgh, Pennsylvania, United States of America; 4 Santa Fe Institute, Santa Fe, New Mexico, United States of America; 5 Department of Materials Science & Engineering, Carnegie Mellon University, Pittsburgh, Pennsylvania, United States of America; Michigan Technological University, UNITED STATES

## Abstract

Additive manufacturing (AM) has rapidly emerged as a disruptive technology to build mechanical parts, enabling increased design complexity, low-cost customization and an ever-increasing range of materials. Yet these capabilities have also created an immense challenge in optimizing the large number of process parameters in order achieve a high-performance part. This is especially true for AM of soft, deformable materials and for liquid-like resins that require experimental printing methods. Here, we developed an expert-guided optimization (EGO) strategy to provide structure in exploring and improving the 3D printing of liquid polydimethylsiloxane (PDMS) elastomer resin. EGO uses three steps, starting first with expert screening to select the parameter space, factors, and factor levels. Second is a hill-climbing algorithm to search the parameter space defined by the expert for the best set of parameters. Third is expert decision making to try new factors or a new parameter space to improve on the best current solution. We applied the algorithm to two calibration objects, a hollow cylinder and a five-sided hollow cube that were evaluated based on a multi-factor scoring system. The optimum print settings were then used to print complex PDMS and epoxy 3D objects, including a twisted vase, water drop, toe, and ear, at a level of detail and fidelity previously not obtained.

## Introduction

Additive manufacturing (AM) can bring digital designs into production quickly and inexpensively compared to traditional manufacturing methods. This has led to widespread adoption in aerospace, automotive and other industries, as well as new applications in soft autonomous robots [1], organ-on-a-chip diagnostic platforms [2], and biological scaffolds for tissue regeneration [3]. Yet these capabilities have also created an immense challenge in optimizing the large number of process parameters in order achieve sufficient print fidelity and part performance. For example, in fused deposition modeling, a 3D digital computer-aided design (CAD) model is converted into a physical object through layer-by-layer deposition of thermoplastic by melting of a solid filament. After deposition, the plastic rapidly cools and serves as support for the next layer, allowing the object to be built from the bottom up. While conceptually straightforward, success requires specific combinations of materials properties and printing process parameters to meet application criteria [4]. The search for the right parameter combination can become even more complex with new materials and new 3D printing methods. For example, silicone elastomers have emerged as a viable material for applications in wearable sensors and medical devices, yet these polymers are often liquids before crosslinking and face gravity-driven collapse using traditional 3D printing. Recently, our group and others have reported new approaches to soft material 3D printing by depositing material within a sacrificial support bath [5–7]. We have termed this process freeform reversible embedding (FRE) [6]. In FRE the support bath is a yield stress fluid; above its yield stress, such as that applied by the nozzle, the bath fluidizes and allows deposition of the printed material. Then, once the stress is relieved with the passage of the nozzle, the support bath resolidifies into a viscoelastic solid, and holds the print in place. After the printing is complete, the liquid polymer is crosslinked and can then be removed from the bath as a free-standing object [6, 7]. However, while the basic FRE process has been demonstrated, the support bath and the soft materials being printed further increases the parameter space of the 3D printing process, thus creating a major optimization challenge.

Several methods have been developed in order to improve the AM process, including topology optimization [8, 9], particle swarm optimization [10, 11], and statistical design of experiments such as the Taguchi method [12, 13]. Optimization using these algorithms can be effective when the selected search space already contains the parameters necessary to achieve good print fidelity. However, adapting these optimization methods for 3D printing of experimental materials, where prior information on the system is often unavailable, can prove difficult. The need for flexibility to change parameters and search space is perhaps why in the practice of AM, the discovery of settings is still largely determined by trial-and-error. Yet, the large space of possible combinations of print settings makes trial-and-error approaches impractical, and the lack of systematization can impede reproducibility. For example, the slicer program that converts a 3D CAD model into machine-readable G-code has about 100 print parameters, such as infill percentage, print speed, deposition rate, and layer height. In a simple case of thermoplastic printing, a factorial design including 5 to 10 main print parameters as factors, each having 5 levels from a possible continuous range, would result in 3,125 to 100,000 possible combinations of settings to print. The combinations of these process settings can increase exponentially for new materials, where the main print parameters are unknown. For example, exploring settings for an experimental material with 20 factors, each with 5 levels, would require approximately three million (20^5^) combinations of print settings. For FRE, materials are 3D printed within a support bath that can be formulated with different chemistries and at different concentrations, further increasing the parameter space.

Here we propose a new expert-guided optimization (EGO) strategy that we test in the context of 3D printing soft materials using FRE. The strategy brings together a structured algorithmic search with flexibility enabled by expert intervention. The EGO approach has three steps that are repeated until a best-case solution is found, given system limitations and the specific application. First is selection of a parameter space as well as the factor and factor levels within that space by an expert (we consider the expert to be someone who has knowledge of the materials and 3D printing, typically the researcher). Second is optimization of factors within the parameter space by a hill-climbing algorithm. Third is selection of a new parameter space by the expert if the hill-climbing algorithm does not yield a solution. The purpose of the expert is two-fold: (i) to select the factors that matter for optimization, based on insight from the field of decision sciences that experts are good at selecting factors important to predictions, yet not as effective in combining the factors [14], and (ii) to escape local maxima by intervening to change the factors or the search space. We chose a hill-climbing algorithm, inspired by previous work used to find drug combinations to kill cancer cells [15]. Alongside EGO, we designed a scoring rubric to quantify print quality that assigns a grade to each response variable (e.g., layer fusion) based on descriptive heuristics. The grades serve to drive the algorithm in real time and provide the opportunity to use low scoring prints as input to improve the process. Here we first apply EGO to 3D print typical calibration objects for thermoplastic filaments, a cylinder and a cube, with polydimethylsiloxane (PDMS) elastomer (Sylgard 184, Dow Corning), a silicone, as the material. We then assess the transferability of the resulting optimum combination of parameters to another experimental material (epoxy) and to complex 3D prints with different shapes and sizes. The results demonstrate that the output from the EGO process can be adapted to other materials, and reinforce its potential as a systematic tool to discover parameters that yield reproducible, high-quality 3D prints.

## Results

### 3D printing silicone elastomers using the EGO strategy

**Fig 1:**
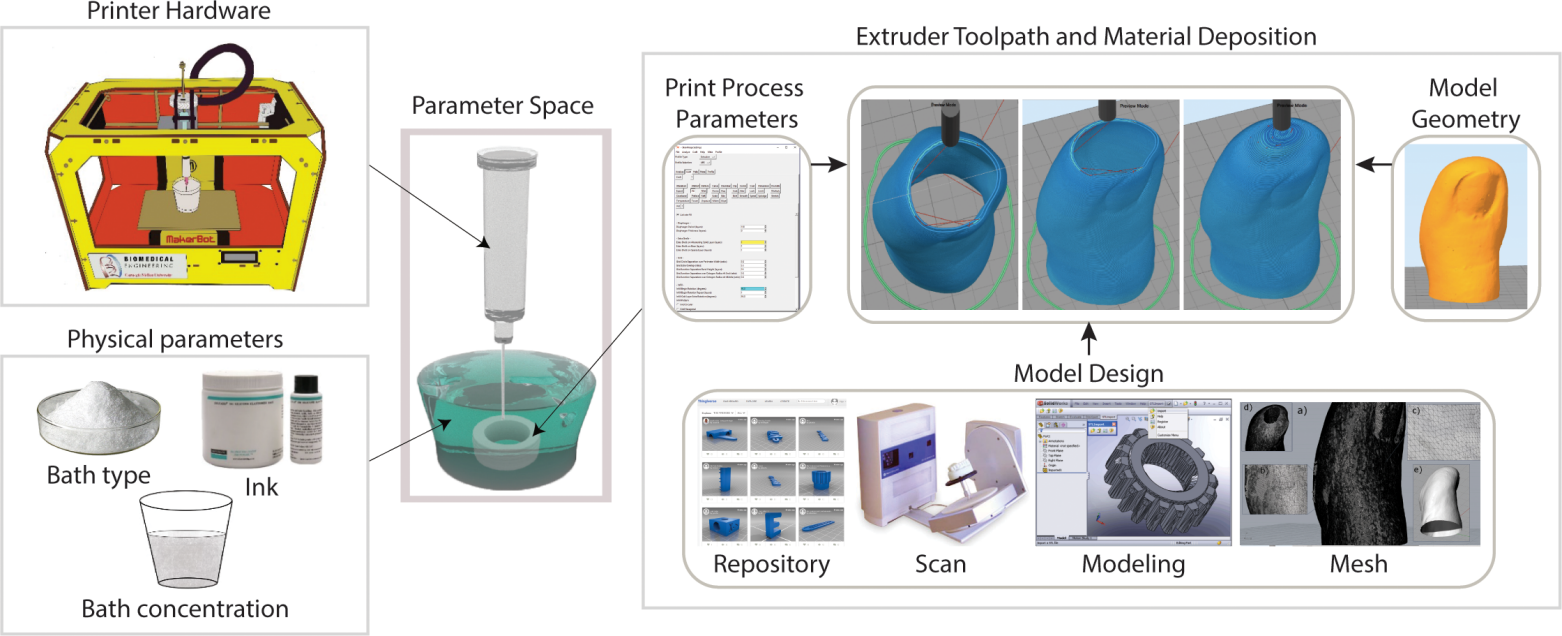
Parameter spaces for 3D printing soft materials using FRE. Five parameter spaces determine the 3D print outcome: physical parameters, printer hardware, model design, model geometry, and print parameters. The model design, model geometry, and print parameters are inputs used by the slicer algorithm to determine the extruder toolpath and material deposition rate. Two of the five parameter spaces (print parameters, physical parameters) are considered throughout the EGO strategy. The printer hardware and model properties (design and geometry) are relatively time intensive to alter quickly and are often preset as design criteria.

**Fig 2:**
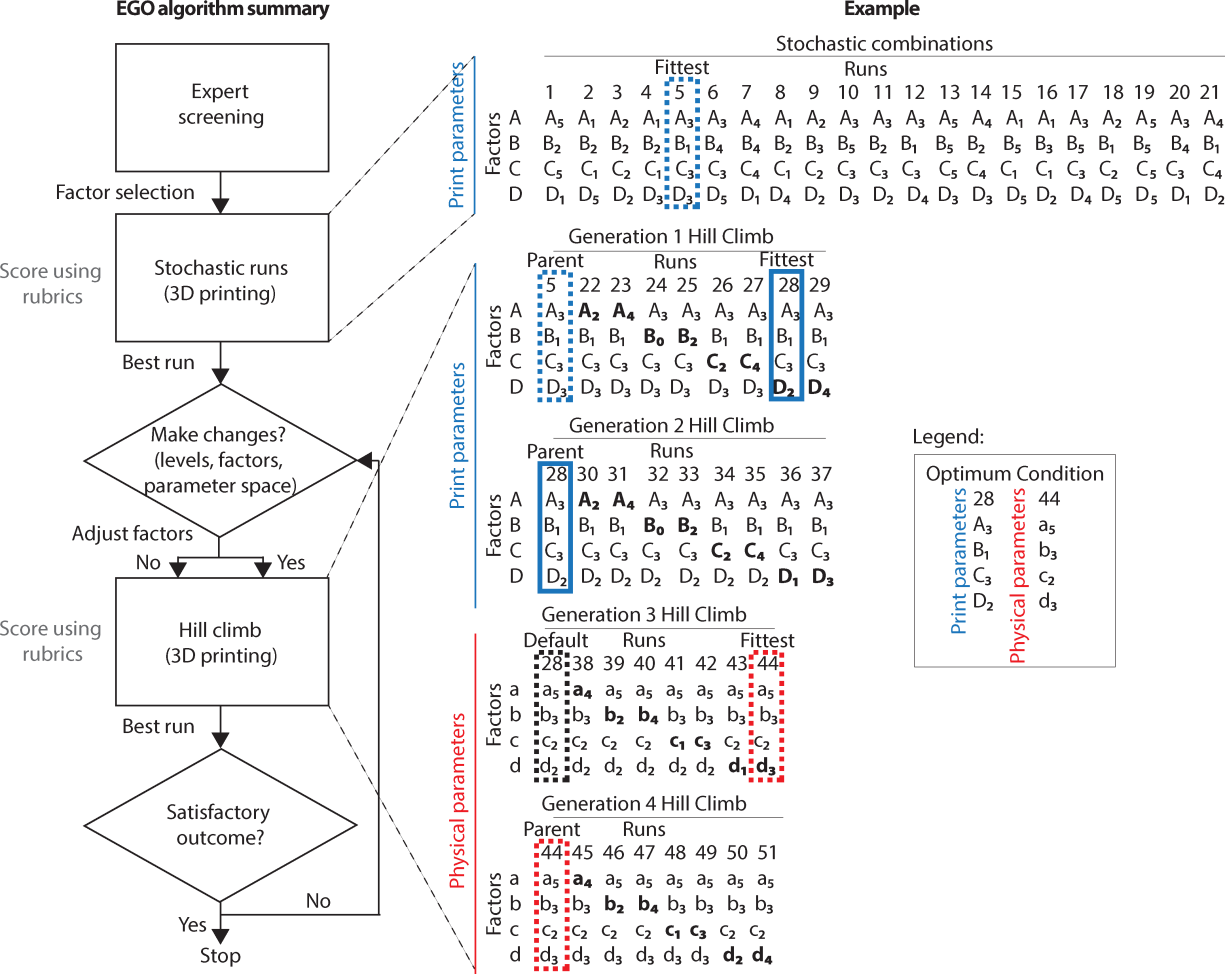
The expert-guided optimization (EGO) strategy. EGO has three steps: (i) Expert screening–identification by the expert of a parameter space (e.g., print parameter space), factors (e.g., A = print speed) and factor levels (e.g., A_1_ = 5 mm/s, A_2_ = 10 mm/s, A_3_ = 15 mm/s, A_4_ = 25 mm/s, A_5_ = 30 mm/s) that matter for print optimization. (ii) Hill-climbing algorithm–includes a stochastic stage and a hill climb stage. The stochastic stage consists of 21 prints made by taking random combinations of factors-levels within the selected parameter space. The hill climb stage uses the highest scoring print (fittest) from the stochastic stage as lead run (parent) for iterative adjustments, seeking a stepwise climb toward the optimum. Hill climbs involve adjustments to one factor-level at a time, to a level above and in a subsequent run, to a level below the parent while the other factors are kept constant at the parent value. (iii) Expert decision-making–changes by the expert if the fittest run from the hill climb does not exceed the parent score. The changes can be to the parameter space (e.g., physical parameters) and/or factors (e.g., a = bath concentration) and/or factor levels (e.g., a_1_ = 0.1 w/v%, A_2_ = 0.4 w/v%, A_3_ = 0.5 w/v%, A_4_ = 1 w/v%, A_5_ = 2 w/v%). The search stops after reaching a full score. The expert may also choose to terminate the optimization if the search is inconclusive after looping between EGO steps (i) and (ii) in attempts to escape local maxima or if the outcome is satisfactory. In the hypothetical example, the stochastic run is performed for four different print parameters (e.g., A, B, C, and D) followed by two generations of hill climb that end in the fittest factor levels for the print parameter space being from run 28 (A_3_, B_1_, C_3_, D_2_). These print parameters are then held constant and third and fourth generation hill climb are performed on the physical parameters (e.g., a, b, c, and d), resulting in the fittest factor levels for the physical parameter space from run 44 (a_5_, b_3_, c_2_, d_3_).

The first step in the EGO strategy is expert screening to select a parameter space, factors, and factor levels. Expert elicitation is common in policy decision-making and has been shown to be important in selecting factors for predictions [14]. The expert in our case is the researcher (in this case the lead author) because of the experimental nature of silicone 3D printing. The parameter space for 3D printing silicone elastomers is broadly divided into five categories (Fig 1); (i) physical parameters (e.g., silicone ink, support bath type, and concentration), (ii) printer hardware (e.g., extruder type), (iii) model design (e.g., factors that determine CAD mesh quality such as CAD production, the source of which is either modeling, repositories, or scans, and post-processing techniques), (iv) CAD model geometry (e.g., size and shape of the object), and (v) print parameters (e.g., extruder speed, layer height). Because the printer hardware is usually fixed and the CAD model design and geometry are often preset, we apply EGO to find the optimum parameter combination for the physical- and print-parameter spaces (Fig 2).

**Fig 3:**
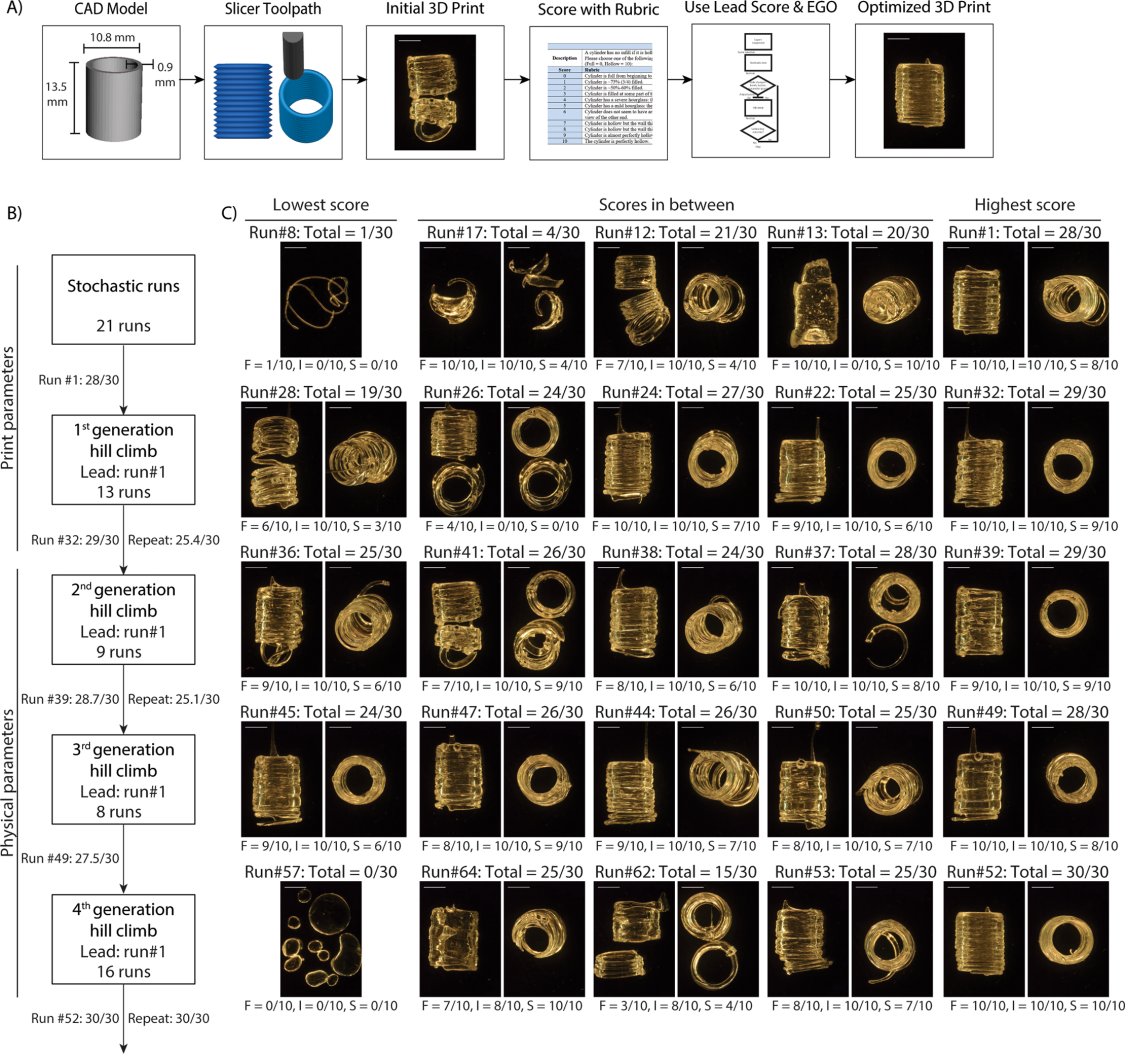
Applying the EGO strategy to 3D printing of calibration cylinders. **(A)** Overview of the steps to optimize the cylinder (CAD model) that is imported in to the slicing software to determine the path of the extruder (slicer toolpath). The resulting product (initial 3D print) is assessed (score with rubric) and the fittest run is used as lead in the hill climb (use lead score and EGO) to reach full score (optimized 3D print). **(B)** Summary of the second step, hill climb, of the EGO strategy for calibration cylinders detailing the total number of runs and the lead run in each within the designated parameter space. **(C)** Representative images of PDMS 3D prints in each generation with the score for each response variables (layer fusion = F, infill = I, stringiness = S) and the total (lowest, highest, in between). Scale bars are 5 mm.

Our first goal was to apply the EGO steps of (i) expert screening, (ii) hill-climbing algorithm and (iii) expert decision-making, to optimize prints of 3D cylinders using silicone elastomer. The overall process is illustrated in Fig 2, highlighting the initial stochastic runs and scoring, followed by repeated hill climbs until the score achieves a satisfactory outcome and EGO is completed. Basic geometric shapes are used for initial testing and calibration in 3D printing because it enables straightforward assessment of the output. Here we selected a hollow cylinder as the starting calibration object (Fig 3A) because the simple shape enables unidirectional continuous circular travel of the extruder, without the acceleration/deceleration associated with corners and other changes in direction. To evaluate print quality, we assigned numeric scores based on qualitative descriptions for three response variables. The three response variables were layer fusion, infill, and stringiness, each with a maximum score of 10, for a total score of 30 for the cylinder (see S1 Fig for examples of scoring and S1–S3 Tables for rubrics). Layer fusion refers to the adhesion between layers throughout the print and is required for mechanical integrity. Infill refers to the presence of material inside the cylinder, which should be hollow, and thus indicates loss of print fidelity. Stringiness is the lack of adhesion between the first few layers of the print.

In the first step of the EGO strategy, the expert selected seven factors and factor levels within the print-parameter space. These factors are Replicator G software inputs that determine the slicing of the CAD model into layers and the printer extruder’s movements, which are defined in the Supplementary Information (S4 Table). First is layer height, which defines the thickness of each layer. Second is speed, which is the rate of extruder movement in the XY plane. Third is filament packing density, which with respect to thermoplastics defines the effective filament diameter inside the pinch wheel, and in more general terms is a means to tweak the flowrate. Fourth is retraction distance, which is the length of filament that is retracted whenever an extruder stop is commanded and its purpose is to control oozing of material. Fifth is solid surface thickness, which are the number of solid layers that are at the bottom, top, plateaus and overhangs. The final two are tower and comb, which change the continuity and starting position of each layer. The expert selected these based on experience with the system, after seeing their impact on prints through factor modifications. It is important to note that not all factors will apply to all print geometries; for example, solid surface thickness refers to horizontal surfaces, which exist in the cube but not the cylinder calibration prints.

In the second step of the EGO strategy, we initialized the overall hill-climbing algorithm by first taking random combinations of these factors, through stochastic runs, and then proceeded to multiple rounds of hill-climb (Fig 2 and Fig 3B). The stochastic runs produced a cylinder with an almost perfect score (28/30), which served as the lead run, where a run is defined as a unique combination of factors and factor levels (see Fig 3C for example results for example stochastic runs and hill-climbs). Next, we performed the 1^st^ generation hill-climb around the lead run, which initially produced a combination with an improved score (29/30). However, repeated prints with the same combination had an average score (n = 5) of 25.4 ± 1.8 (SD), demonstrating that this factor-level combination did not produce a consistent output better than the lead run. We next searched factors specific to the physical-parameter space, which were support bath pH and stirring time, PDMS base-to-catalyst ratio and PDMS curing time after printing (S5 Table). We initiated a 2^nd^ generation hill-climb by varying the levels of these factors within the physical-parameter space while fixing the print-parameter factors to values from the initial lead run. The highest score was 28.7/30, which when repeated had an average score (n = 12) of 25.1 ± 1.4 (SD), indicating that the hill climb across the physical-parameter space did not improve the score. However, a higher score was achieved with a specific PDMS base-to-catalyst ratio, suggesting this may be an important factor. A 3^rd^ generation hill climb varying only the PDMS base-to-catalyst ratio was performed, but also did not improve the score beyond the initial lead run (highest score 27.5/30). The expert then chose a wider range of physical parameters for the 4^th^ generation hill climb, specifically the type of PDMS, the specific type of the support bath Carbomer, and the support bath concentration. This resulted in a set of factor-levels that maximized the quality of the resulting cylinder (30/30), which when repeated had an average score of 30 (n = 8). In brief, the cylinder was optimized after four generations of hill climb (Fig 3B), requiring a total of 67 runs. S2 Fig provides a close-up of example prints with low, medium, and full score prints.

**Fig 4:**
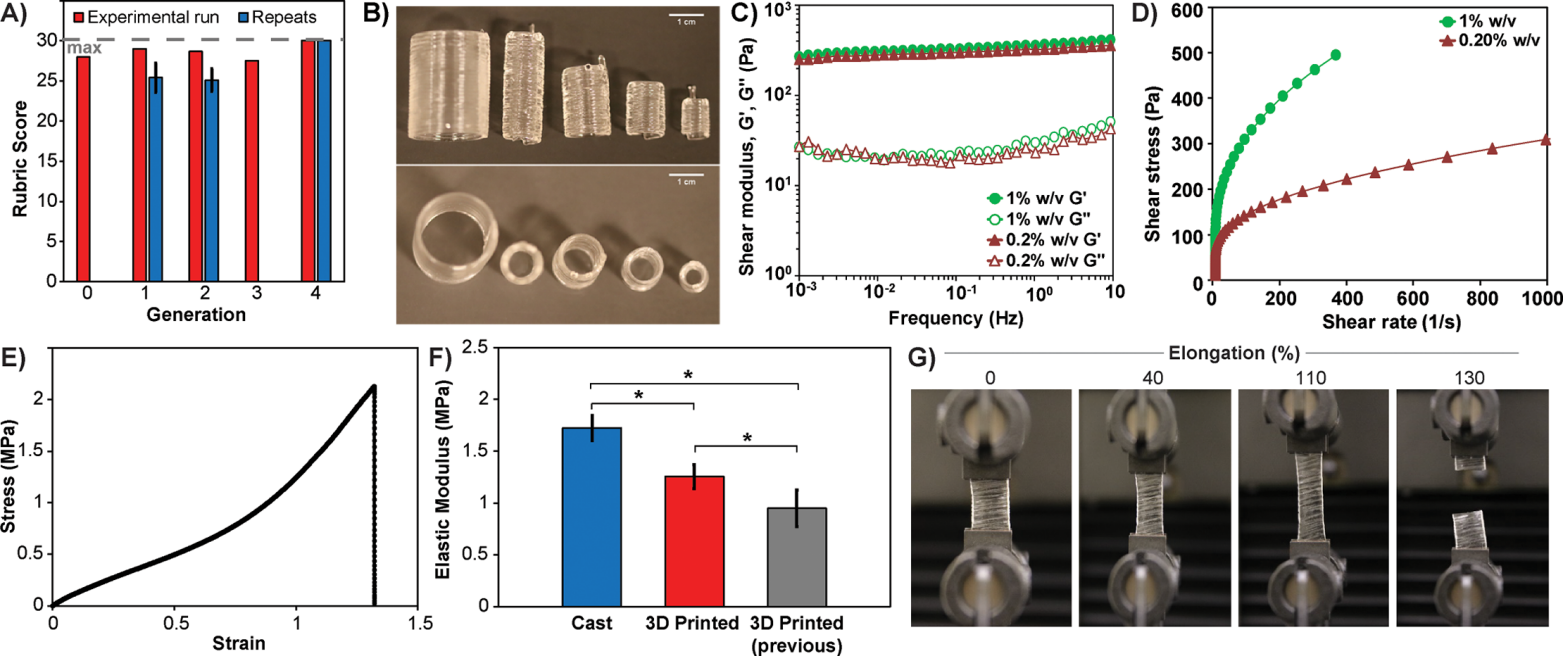
Characterization of the cylinder optimization conditions and resulting mechanical properties. **(A)** Summary of the EGO strategy applied to optimize the cylinder showing the highest score from each generation and the target score of 30. The cylinder optimized after four generations of hill climb. **(B)** PDMS 3D printed using the EGO optimum scaled-up to five different sizes. The cylinder used throughout the EGO strategy is the second cylinder from the right. Scale bar is 1 cm. **(C)** Shear modulus as a function of frequency across the 1% w/v (starting) and 0.2% w/v (optimum) Carbopol 940 bath concentrations. The baths are dominantly viscoelastic solids with the storage (G’) modulus larger than the loss modulus (G”). **(D)** Shear stress versus shear rate at 1% w/v (starting) and 0.2% w/v (optimum) Carbopol 940 bath concentration with a yield stress of 140 Pa and 70 Pa yield stress, respectively. The yield stress is the shear stress at ~4.5 s^-1^ shear rate (the y-intercept for the linear portion of the shear stress versus shear rate curve). **(E)** Representative stress-strain curve showing the 3D printed PDMS strip subject to uniaxial tensile tests with the inset displaying the region that is linearly elastic, y = 1.2x + 0.0042 (R^2^ = 0.996), up to 10% strain. **(F)** The mean elastic modulus was 1.7 ± 0.1 MPa (SD) for monolithic PDMS (n = 6) made by casting in a previous study [17], 1.2 ± 0.1 MPa (SD) for 3D printed using the EGO found optimum (n = 5), and 0.95 ± 0.2 MPa (SD) for 3D printed (n = 8) in a previous study [6]. Both cast PDMS and 3D printed PDMS have the same base-to-catalyst ratio of 10:1. **(G)** Representative images of 3D printed PDMS across uniaxial tensile strains up to 130% elongation at break.

As noted, the 4^th^ generation hill-climb produced optimized parameters that achieved a maximum score of 30 that was maintained upon repeated prints (Fig 4A). These parameters worked not only for the initial calibration cylinder, but also enabled scaling of the cylinder from 8 mm × 10 mm up to 21.9 mm × 27.3 mm (diameter × height), with comparable fidelity (Fig 4B). It was found that the concentration of Carbopol 940 used for the support bath was an important parameter. The optimum parameters used a bath concentration of 0.2% w/v, rather than the initial 1% w/v. Rheological analysis showed that both baths behaved as viscoelastic solids, with difference of ~10% in storage modulus (Fig 4C) of the optimum bath (G’_0.2% w/v_ ~ 319 Pa) versus the original support (G’_1% w/v_ ~ 360 Pa). Additionally, the optimum bath at 0.2% w/v concentration had a yield stress of 70 Pa, approximately half that of the 1% w/v bath at 140 Pa (Fig 4D). We also used uniaxial tensile testing to determine whether the EGO strategy improved the mechanical properties of the 3D printed PDMS. A representative stress versus strain curve shows that the 3D printed PDMS behaved as a linearly elastic material almost up until failure (Fig 4E). The elastic modulus of the optimized 3D PDMS prints (n = 5) was 1.2 ± 0.1 MPa (SD), which is comparable to cast PDMS [16, 17] and significantly better than previously reported values for 3D printed PDMS [6] (Fig 4F). We also examined elongation to break of the 3D printed PDMS, and compared this to the cast PDMS, which has an elongation to break of 140% [18], representing the maximum value we could theoretically obtain. Considering that 3D printed polymers are known to have lower elongation to break between printed layers [19], our results demonstrate that we can minimize this issue for PDMS using the EGO strategy. The 3D printed PDMS had an elongation to break that ranged from 80% to 110% (Fig 4G). While this is less than cast PDMS, it demonstrates very good layer-to-layer adhesion of the 3D printed PDMS, achieving relatively large deformations without failure. In total, the mechanical characterization demonstrates that the EGO strategy can produce 3D printed PDMS parts with properties similar to cast PDMS.

### Using the EGO strategy to 3D print objects with increased structural complexity

**Fig 5:**
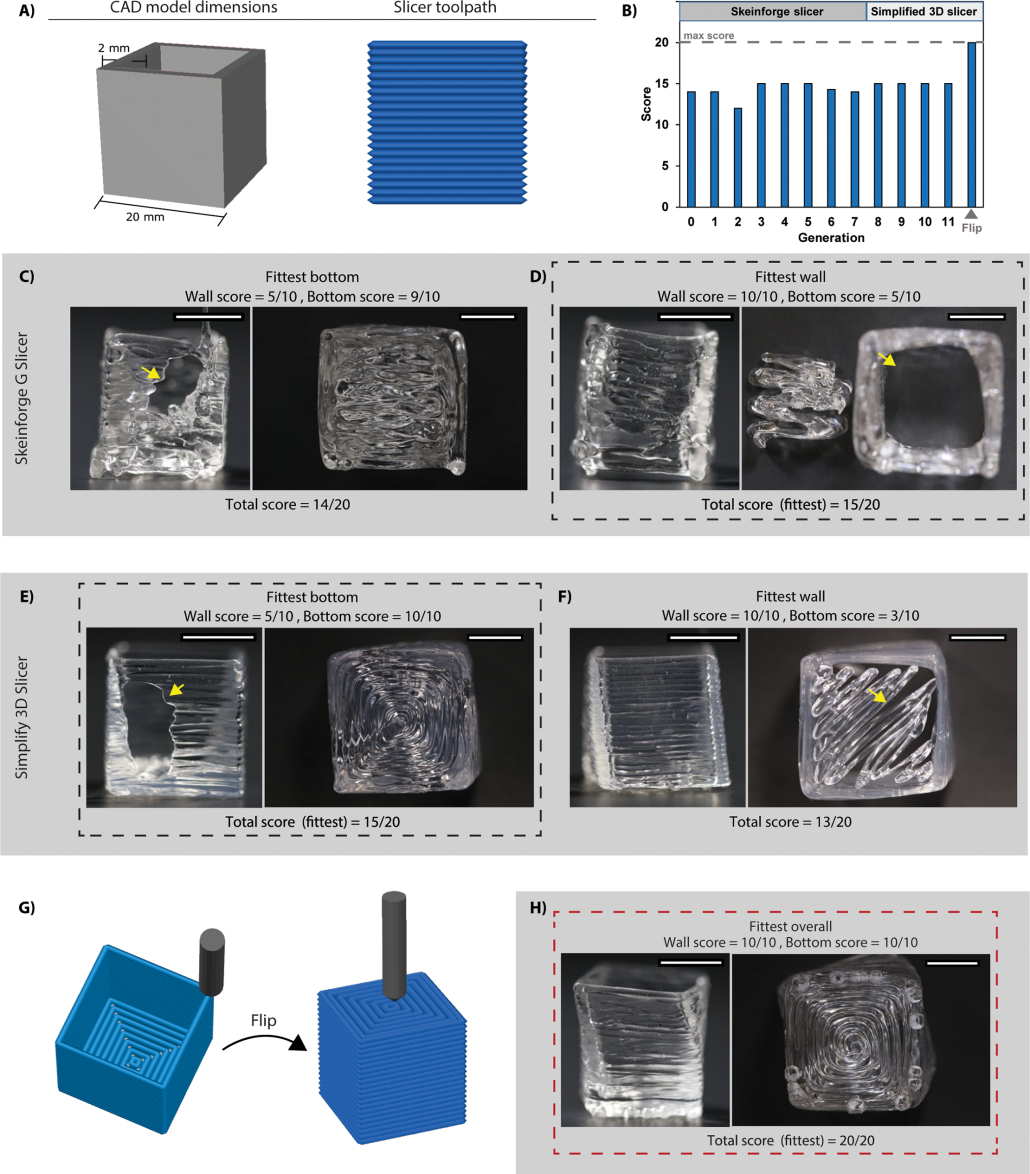
Application of the EGO strategy to 3D print calibration cubes. **(A)** Dimensions of the cube CAD model, 20 mm × 20 mm × 20 mm, L×W×H, and the resulting S3D slicer toolpath. **(B)** Summary of the EGO strategy applied to optimize the cube showing the highest score from each generation and the target score of 20. The cube optimized after 12 generations of hill climb. **(C)** Images of the PDMS 3D print using the Skeinforge CAD slicer to determine toolpath. The print with high bottom score (9/10) had poor wall score (5/10). **(D)** Images of the PDMS 3D print made using the Skeinforge CAD slicer to determine toolpath. The print with poor bottom score (5/10) had full wall score (10/10), and had the fittest total score (15/20). **(E)** Images of the PDMS 3D print made using the S3D CAD slicer to determine toolpath. The print with full bottom score (10/10) had poor wall score (5/10), and had the fittest total score (15/20). **(F)** Images of the PDMS 3D print made using the S3D CAD slicer to determine toolpath. The print with poor bottom score (3/10) had a full wall score (10/10). **(G)** S3D slicer toolpath of the cube before and after the flip, inverting the build orientation for 3D printing in the support bath, which was achieved at the 12^th^ generation of the cube hill climb. **(H)** Image of PDMS 3D prints made using the S3D CAD slicer to determine the toolpath. A full total score (20/20) was reached after the flip. Scale bars are 1 cm.

We next applied the EGO strategy to 3D prints with more complex geometry. The cylinder had no corners and only vertical surfaces (walls). We selected a 5-sided hollow cube with 4 sides and a bottom (Fig 5A), which added 90-degree angles within each layer and intersecting horizontal and vertical surfaces. We had previously reported that horizontal surfaces could not be reliably printed with PDMS using the FRE process based on our trial-and-error attempts [6]. The cube scoring rubrics included two response variables, wall fusion and bottom surface (S6 and S7 Tables and S1 Fig), which each had a maximum score of 10, for a total score of 20. As expected, printing the cube proved more challenging than the cylinder, and required 12 generations of hill climb to fully optimize (Fig 5B). In the first EGO step, the expert selected the same print-parameter factors and factor levels that were used for the cylinder. In the second EGO step, we took random combinations of these factor-levels for the stochastic runs, as used for the cylinder, and initialized the overall hill-climbing algorithm (S3 Fig). The next four generations of hill climb attempted to improve the score, but the lead runs from each never exceeded a score of 15/20. Because the score did not improve, the expert attempted to break away from a structured hill climb by selecting factor-levels based on the expert’s best guess. This 5^th^ generation hill climb produced a lead score of 15/20, and thus also did not improve upon the previous hill climbs. A recurring problem was the bottom surface (base) of the cube, which printed poorly. The expert continued with a 6^th^ hill climb on the print parameter space with additional factors (e.g., infill pattern, grid extra overlap, and infill solidity) that pertain to changes to the bottom surface of the 3D print (S4 Table). However, the resulting score (S3 Fig) was worse than the previous lead run (15/20) identified in the 4^th^ generation hill climb. Insofar, the hill climbs were taking place on the print parameter space with no improvement, a 7^th^ generation hill climb was initiated, this time on the physical parameter space. The changes were similar to those from the 4^th^ generation hill climb of the cylinder, namely in modifying the type of silicone, the support bath Carbomer, and the support bath concentration. However, these changes produced comparable scores as before (15/20), and did not improve upon the lead run from the 4^th^ generation hill climb. We identified the problem to be that the wall and the bottom could not both be printed with good fidelity with the same parameters, a perfect print score of either one resulted in a poor print score of the other (Fig 5C and 5D).

The expert then re-initiated the EGO strategy for the cube using another CAD slicing program, specifically Simplify 3D (S3D). Since each slicer uses a different algorithm to convert the CAD model into a toolpath, this switch may change the extruder motion and improve printing of the cube base. However, three generations of successive hill climbs resulted in the same score (15/20) as the lead run obtained from earlier hill climbs using Skeinforge, the slicer in Replicator G. Thus, while using S3D changed the toolpath, the same problem remained where we could not print both the bottom and wall surface in one cube (Fig 5E and 5F). At this point we (the expert) stopped performing hill climbs, having reached 167 print attempts (Skeinforge, n = 110 prints; S3D, n = 57 prints) with the potential of having reached a global maximum for this difficult to print structure.

The failure of the EGO process to improve the cube score above 15/20 while changing print parameters, physical parameters and slicing software highlights the challenge of printing soft materials. However, we did achieve a full cube score (20/20) by having the expert select a parameter space that was not initially considered to be important at the start of the EGO process. Specifically, we identified that build orientation might be important when printing because of the wall defect that appeared in some prints (Fig 5B and 5C), and reasoned that printing the horizontal surface last, rather than first, might eliminate this issue. Unique to FRE printing is that we can select orientations that would be challenging or impossible to print in air due to the need for excessive printed support material. In addition, a previous study had focused on build orientation as a means to improve the surface roughness of prints [20]. In our case, rotating the cube 180-degrees so that it was upside down (Fig 5G) resulted in the highest scoring cube (20/20) (Fig 5H). This highlights the importance of build orientation as an important parameter space and the flexibility that the EGO strategy provides through expert input. However, the flipped cube was not perfect, as it contained a slight shift between layers that gave it a twisted appearance. The rubric did not account for this, which illustrates the importance of the scoring system and its ability to fully assess print output.

### Using the EGO output to 3D print different materials and geometries

**Fig 6:**
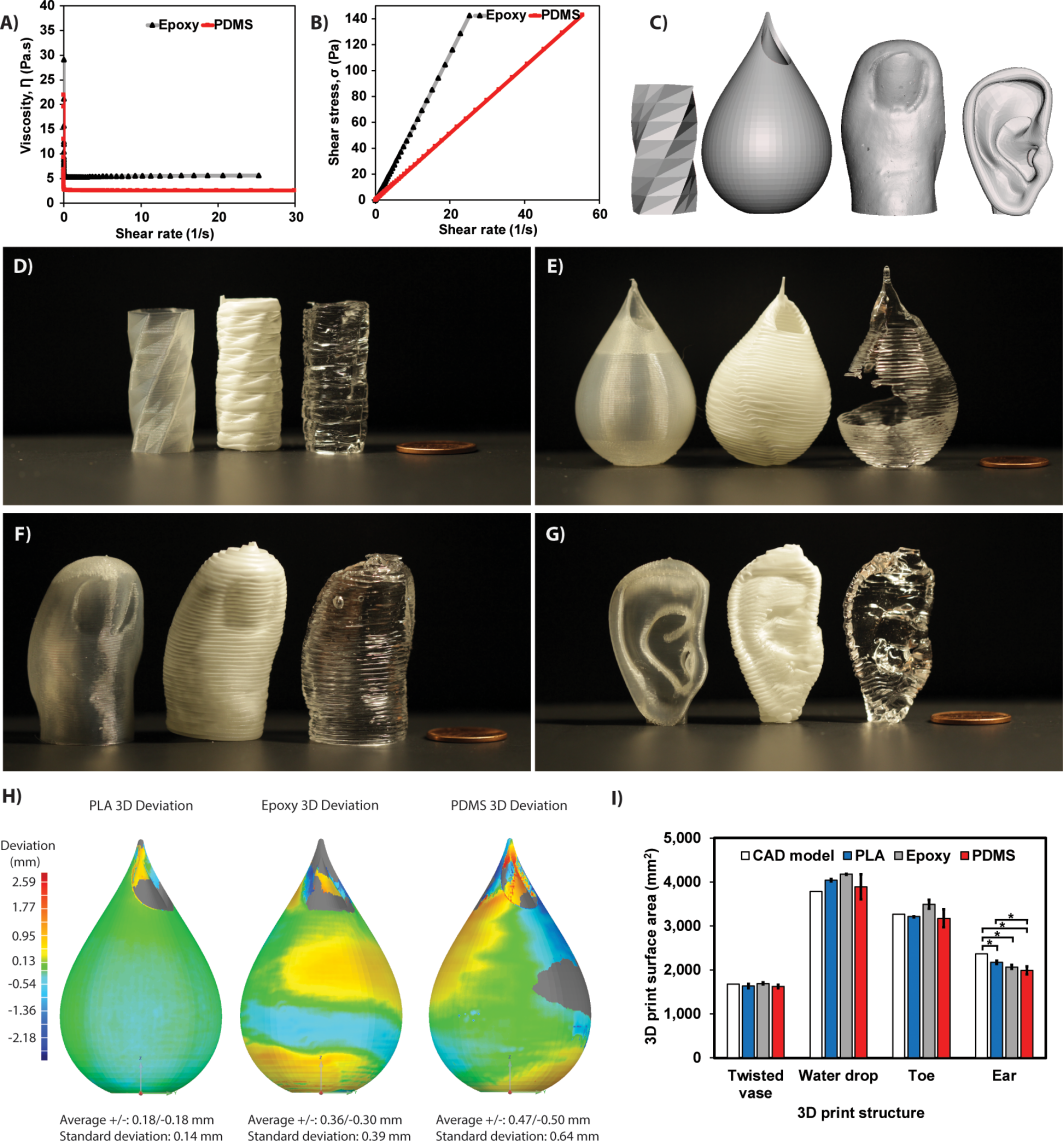
Using the EGO found optimum to 3D print epoxy and PDMS in complex geometries. **(A)** The viscosity of both PDMS (~3 Pa·s) and epoxy (~5.3 Pa·s) is constant as a function of shear rate. **(B)** The linear increase of shear stress as a function of shear rate confirms that before the crosslinking step, both PDMS and epoxy inks are Newtonian fluids. **(C)** CAD models of the different geometries used for 3D printing with both experimental (PDMS, epoxy) and standard (PLA) materials. **(D)** Representative image of a twisted vase (15 mm × 33 mm, W×H) 3D prints with standard PLA (left), epoxy (middle), and PDMS (right). **(E)** Representative image of a water drop vase (17 mm × 52 mm, W×H) 3D prints with standard PLA (left), epoxy (middle), and PDMS (right). **(F)** Representative image of a life-size toe (25 mm × 44 mm, W×H) 3D prints with standard PLA (left), epoxy (middle), and PDMS (right). **(G)** Representative image of an ear (20 × 35 mm, W×H) 3D prints with standard PLA (left), epoxy (middle), and PDMS (right). **(H)** Representative color maps of the water drop vase highlighting the deviation of PLA, epoxy, and PDMS 3D prints from the CAD model. **(I)** Surface area of the CAD model versus the PLA control, epoxy and PDMS for each 3D printed structure (n = 3) shown in D–G.

Finally, we sought to determine the extent to which the parameter settings identified with the EGO strategy for PDMS could be used to 3D print other materials and geometries. Specifically, epoxy was selected because it is a thermoset that when crosslinked has significantly different mechanical properties from PDMS elastomer, but as a prepolymer can have similar rheology. Epoxy has been 3D printed before [21], but with nano-clay platelets that resulted in a viscoelastic ink with four-fold increase in viscosity over the pure resin. An epoxy resin without additives, remaining a Newtonian fluid and of low viscosity has not been previously 3D printed, and thus would be a major achievement with potential industrial applications. To select an appropriate epoxy we performed rheological analysis and found that the viscosity of the Sylgard 184 PDMS was ~3 Pa·s. Based on this we selected a widely available industrial-grade epoxy, Epon 828 base and Epikure 3200 curing agent, that had a similar viscosity of ~5.3 Pa·s (Fig 6A). In addition, both the PDMS and the epoxy showed a linear increase of shear stress with shear rate (Fig 6B), establishing that these behave as Newtonian fluids prior to crosslinking.

To assess printability, we selected CAD models of different shapes that included a twisted vase, a water drop vase, a human toe and a human ear (Fig 6C). We used the print settings identified as the optimum for the cube with the S3D software. As a control and for comparison, each 3D shape was also printed in PLA using a MakerBot Replicator 3D printer equipped with the standard thermoplastic extruder. Results show that the EGO optimum is transferable between PDMS and epoxy inks and across widely varying print geometries (Fig 6D–6G). The twisted vase provides a good example of achieving comparable print quality across material types (Fig 6D). The water drop vase showed the ability to print the high aspect ratio portion by the top opening (Fig 6E), however, the PDMS print demonstrated the same problem with wall defects observed for the calibration cube (Fig 5E). The anatomical models of the toe (Fig 6F) and the ear (Fig 6G) confirmed the ability to print more complex structures that lacked the symmetry of the calibration objects and different vases.

Next, geometry for each material print was quantified using a 3D scanner to assess fidelity. Specifically, this enabled us to determine deviation of the printed structure to the original 3D CAD model. Representative 3D images of spatial deviation for the water drop vase (Fig 6H) show that the PLA print aligned well with the original CAD model, while the epoxy and PDMS prints showed 2–3 times more spatial deviation (n = 3 per material). Still, the average deviation was less than ±0.5 mm for all materials. As noted in the photograph (Fig 6E), the PDMS water drop had a wall defect that was material specific, as it did not occur for the epoxy. Finally, we used surface area as a metric for comparison across print geometries and showed that in general the EGO optimized print setting was comparable to the original CAD model and PLA prints (Fig 6I). Only the ear 3D prints showed statistically significant differences in surface area between material types and the CAD model, and in this case even the PLA print had a statistically significant difference. This demonstrates the role that the geometry of the CAD model plays in print fidelity, and reinforces our inclusion of geometry in the parameter space for optimization.

## Discussion

In practice, the optimal settings for 3D printing are still largely found by trial-and-error, which may be time consuming and inefficient. Interestingly, insights from decision science have shown that experts are good at selecting factors that matter in prediction but not as good at combining those factors to make a prediction [14]. We take advantage of this insight and draw on expert knowledge to identify the factors that matter in printing and let an algorithm decide how to combine those factors. Interventions from the experimenter also provide an opportunity to escape local maxima, as an alternative to randomness used in other approaches such as simulated annealing [22]. Thus, unlike trial-and-error experiments that implicitly rely on the experimenter’s judgment, EGO makes expert judgment an explicit input that is enhanced by algorithmic search. Many possible algorithms can be used with this approach. Here, we use a hill-climbing algorithm based on previous work used to find drug combinations to kill cancer cells [15]. Similar approaches have been used to combine molecules to support stem cells in culture [23]. Evolutionary algorithms have also been employed in the context of AM. Without expert intervention to explore search spaces, the applications have focused on specific areas without seeking to improve other aspects. Examples range from optimizing the CAD mesh triangle layout in the design software to directing the CAD slicing program to find the optimum layer height given the model geometry [24].

Overall, the EGO strategy provides an effective approach to optimize 3D prints using the FRE process where the bath properties, ink properties and print process settings comprise a large parameter space. The key achievement of the EGO strategy was to optimize the 3D printing of a range of objects using the FRE process with extremely challenging materials such as PDMS elastomer and epoxy. Other groups have 3D printed a few select types of silicones and epoxies [7, 21], but these were modified to be shear thinning or rapidly photocrosslinked to improve printability. In contrast, here we show that low viscosity, Newtonian fluids can be 3D printed using FRE, and that the EGO strategy provides a systematic approach to print complex parts and improve mechanical performance. For example, PDMS prints made with the EGO optimum had elongation to break of ~100% and an elastic modulus of ~1 MPa (Fig 4F and 4G), comparable to cast PDMS [16, 17]. This is important because 3D printing using extrusion based methods often leaves void space that reduces mechanical integrity, but our results clearly show that this can be minimized using the EGO strategy.

While not based on first principles, the results of the EGO strategy performed here do provide useful insight into the potential interactions driving print fidelity. For example, the calibration cube had a persistent vertical wall defect for prints that had a good horizontal (XY) bottom surface (Fig 5C and 5E). This wall defect was eliminated when the bottom surface was discontinuous (Fig 5D and 5F), strongly suggesting that the wall defect arose because of the flow of PDMS from the wall to the bottom surface. The wall defect was also observed for the water drop vase, which like the cube had a horizontal base surface. Inverted, the cube reached a full score (20/20) with the same parameter settings used to achieve the non-inverted cube that had a lower score (15/20). These observations suggest a few different possibilities related to the printed materials and the support bath. The weight of the volume of support bath on the bottom surface can exert a downward force on the vertical walls, which are still liquid, and lead to wall collapse. Similarly, the liquid PDMS in the vertical walls can also be pulled by gravity and collect in the bottom surface. The wall defect can also be driven by surface energy minimization as the immiscible, hydrophobic, PDMS minimizes contact area with the hydrophilic support bath. While testing these possibilities is beyond the scope of the current work, the wall defect behavior is likely due to a combination of these factors. It is important to highlight that the EGO process used expert-guided change in print orientation to resolve the wall defect in the cube. The expert could have also made other changes. For example, the PDMS could have been modified so as not to flow after deposition, through immediate crosslinking such as by UV curing, or use of a rheological modifier. However, we notably achieved high-quality print output without modifying the nature of the PDMS or epoxy material, and instead used the EGO process to optimize other factors in the overall parameter space. Thus, we expect that EGO can have wide application in optimizing new 3D printing processes, especially for non-traditional materials.

Looking towards future applications, we see the EGO strategy to be well suited to optimize the 3D printing process across a wide variety of techniques, including fused deposition modeling, stereolithography and powder bed-based AM processes. For FRE printing, it should enable the use of multiple materials with disparate chemistries and properties, such as printing epoxy and PDMS within the same overall structure. For the related freeform reversible embedding of suspended hydrogels (FRESH) technique our lab has developed [3], it should improve our ability to 3D bioprint functional tissues, where we can include cell type, growth factor levels and other biological properties as part of an expanded parameter space. Finally, after performing EGO the results may actually provide insight into how the various parameters dictate print fidelity, and which ones dominate under certain conditions. For example, for the 5-sided cube we identified print orientation to be critically important and it enabled an increase in the score from 15 to 20. In addition to demonstrating that print orientation can be as important in EGO as it is in other AM techniques, it also shows how leveraging the expert to jump to a new parameter space is unique to EGO as compared to other optimization methods, and can provide major improvements to the fidelity of 3D printed parts.

## Materials and methods

### The expert-guided optimization (EGO) strategy

The first step of the EGO strategy (Fig 2, Expert screening) uses an expert (first author S. Abdollahi) to identify factors, and respective factor levels, in a parameter space likely to have the highest impact on the 3D print. The second hill climb step includes an initial stochastic search followed by a hill climb. For the stochastic search, 20 random combinations of each factor’s levels are chosen (Fig 2, Stochastic runs). The expert also selects a "best guess" that reflects the combination expected to have the highest score. The structure is then printed 21 times using the random combinations plus the best guess parameters. The quality of the resulting 3D prints are then quantified using the scoring rubrics for the response criteria (fusion, stringiness, and infill), which are further detailed in the *Response variables and scoring rubrics* section below and in the Supplementary Information (S1–S3, S6 and S7 Tables, S1 Fig). The highest scoring run is the fittest combination, called the parent, and is used as a seed for the hill climb.

In the hill-climb phase (Fig 2, Hill climb), the parent from the previous generation is modified by local adjustments to the levels of each of its factors, one at a time. For example, one run would be identical to the parent but with a higher printing speed. Another would be identical to the parent but with a lower retraction distance. These local adjustments are then printed and scored. If the highest score from this generation is greater than the highest score from the previous generation, the print with the highest score is taken to be the new parent, and the process is repeated. If there is no improvement, the expert selects new factors, and the hill-climb phase is repeated with those new factors. These new factors can be on another parameter space, for example varying the ink base to catalyst ratio in the physical-parameter space rather than the layer height in the print-parameter space. When the expert decides to change the parameter space, factors on the original parameter space are kept constant at the fittest combination. Thus, EGO ends with a set of fittest parameter combinations for all parameter spaces. A detailed definition of each factor and the respective levels chosen by the expert are provided in the Supplementary Information (S4, S5 and S8 Tables).

### Response variables & scoring rubrics

To quantify the quality of 3D prints, the characteristics of an optimal 3D print were defined for two simple objects: a cylinder and a cube. For each structure, we constructed criteria (response variables) for determining whether the print was optimal. For the cylinders, the criteria were: (i) layer fusion–adhesion between printed layers, (ii) stringiness–adhesion of the first layers that often overhang like strings despite layer fusion in the bulk print, and (iii) infill–the preservation of hollow space in the center of the cylinder, rather than bulging in of the material into this space. For the cubes, we used two criteria: (i) layer fusion and (ii) bottom–referring to the integrity of the cube’s base. Consequently, a three-variable and a two-variable scoring system were developed (S1–S3, S6 and S7 Tables) to assess 3D print outcomes for the cylinder and cube, respectively. In this system, the experimenter uses a rubric and grades the structure blind, without knowledge of the parameters used to make the print. The scoring system provides a means to extract information from poor prints that are hard to quantitatively characterize (e.g., by measuring their dimensions), to make the next set of decisions. The rubric is specific to the print (e.g., cube) and provides a range of scores with matching descriptions that define the quality levels of the response variable (e.g., layer fusion). Each response variable is assigned a score from 0 to 10. A simple example is to assign a score of 0/10 for layer fusion of a printed cube with no apparent structure, 5/10 for the fusion of one sidewall, and 10/10 for layer adhesion of all four sides (S1 Fig). The total print score is the sum of the response variable scores for a given print.

### 3D printing: Experimental setup, ink, and support bath preparation

3D printing silicone elastomers is based on the FRE printing process [6] that extrudes the pre-polymer in a sacrificial support bath to hold the liquid layers during printing. The FRE process involves subsequent crosslinking of the liquid ink, which is accelerated by heat curing, and the print is then removed from the support bath. A Makerbot Replicator^TM^ dual 3D printer (MakerBot Industries, LLC, Brooklyn, NY) was modified as described previously for liquid extrusion, with the printer’s original thermoplastic extruder replaced with a syringe-based extruder [3]. The G-code for the extruder’s path for depositing the ink layer-by-layer is based on a CAD model that is processed into layers using a slicer program. Both an open source slicer (Skeinforge) and a commercially available slicer (S3D) were used, with the extruder toolpath based on the algorithm inherent to each program, model geometry, and user inputs. The initial printing speed was 10 mm/s and varied for different runs throughout the application of EGO. The digital CAD model of the cube, cylinder, ear, twisted vase, and water drop designs were obtained from the Thingiverse (www.thingiverse.com) repository. The toe CAD model was developed from a scan of a life-size ceramic toe cast of an actual toe using the NextEngine Desktop 3D scanner (NextEngine, Inc., Santa Monica, CA).

The PDMS and epoxy inks used for extrusion must first be prepared before 3D printing. The Sylgard 184 (Dow Corning Corporation, Midland, MI) was prepared by mixing the two-part, base-to-catalyst curing agent at a 10:1 ratio by weight. Other elastomeric materials used in the physical-parameter space were Sylgard 186, Sylgard 567, and Dow Corning 3–4241 (Dow Corning Corporation, Midland, MI) at 10:1, 1:1, and 1:1 base-to-catalyst ratio by weight, respectively. The ink components were mixed in a Thinky-Conditioning planetary centrifugal mixer (Phoenix Equipment Inc, Rochester, NY) for 2 minutes, and then defoamed for 2 minutes, both at 2000 RPM. The epoxy ink base, Epon 828 (Hexion, Momentive Specialty Chemicals, Inc., Columbus, OH), and catalyst-curing agent, Epikure 3200 (Hexion), were similarly mixed at 5:1 base-to-catalyst ratio by weight. The inks are used to fill a 10 ml plastic syringe to mount onto the printer. The syringe nozzle was an 18 gauge blunt needle tip (Jensen Global Inc, Santa Barbara, CA) with 0.965 mm inside diameter (ID). Other needle extrusion tips used in the physical-parameter space were 24 gauge (0.381 mm ID), 23 gauge (0.406 mm ID), 17 gauge (1.219 mm ID), 16 gauge (1.346 mm ID), and 14 gauge (1.75 mm ID). Tapered needle tips were also used, consisting of 25 gauge (0.28 mm ID), 20 gauge (0.63 mm ID), 18 gauge (0.84 mm ID), 16 gauge (1.2 mm ID), and 14 gauge (1.52 mm ID).

The support bath for 3D printing the ink was prepared with polyacrylic acid microgels, referred to as Carbomer (Lubrizol Corporation, Wickliffe, OH). The Carbomer was first hydrated (pH~3) by mixing it with deionized water for ~20 minutes, and subsequently neutralized (pH~7) with the addition of sodium hydroxide (NaOH) to swell the microgels. The physical parameter space included different Carbomer types and different concentrations of the suspension. Carbopol 940 was made at 0.1% w/v, 0.2% w/v, 0.5% w/v, 1% w/v, and 2% w/v. ETD 2020 was made at 0.2% w/v and 1% w/v, and all other polymers (Carbopol 974, Ultrez 10, Carbopol 934, Carbopol 1342, Carbopol 941, Pemulen 1621) were prepared at 1% w/v. The bath suspension was mixed and defoamed in the Thinky-Conditioning mixer for 2 minutes each at 2000 RPM. For FRE 3D printing, the tip of the syringe extruder tip was lowered into the Carbomer support bath container at the center of the build platform. After 3D print completion, the container with embedded print was placed in an oven overnight at 65 ^o^C for the PDMS ink to crosslink. The resulting 3D print was removed from the bath and washed under running water to remove leftover Carbomer, after which it was dried and analyzed.

### Rheological characterization

Rheological analysis of the Sylgard 184 silicone elastomer, Epon epoxy and Carbomer support baths was conducted using a Bohlin Instruments Gemini 200 Rheometer (Malvern Instruments Ltd, UK) with a cone and plate geometry (4^o^ cone angle, 40 mm diameter) and a 150 μm gap size. The unidirectional shear stress and viscosity were measured as a function of shear rate in the 0.005 Pa to 500 Pa range. Experiments were performed under isothermal conditions (25 ^o^C).

### Mechanical characterization

To create strips made from 3D printed PDMS, cuts were made with scissors parallel to the PDMS cylinder wall. Uniaxial tensile testing of PDMS strips (average size: 1.7 mm × 10.9 mm × 13.8 mm) was performed using an Instron 5943 (Instron, Norwood, MA). A total of 6 samples were stretched at a rate of 2 mm/min until failure. The elastic modulus of the 3D printed PDMS was determined from the linear elastic region of the stress-strain curve, up to strain values of 10%. Young’s (elastic) modulus was obtained through Hooke’s law, E = σ/ε, in which σ is the applied stress and ε is the strain. The elongation at break was ε_b_ = L_b_/L_o_, where L_b_ is the maximum length before break. Tests were conducted at ambient temperature.

### Imaging

A Nikon SMZ1500 stereomicroscope (Nikon Inc., Mellville, NY) with a 1X objective was used for imaging cylinders with a mounted Nikon D5100 digital single-lens reflex (DSLR) camera. Cylinder images were processed with Image J (National Institutes of Health, Bethesda, MD). The cubes and other exploratory prints were imaged using a standard Canon Rebel T6i and T3i DSLR cameras with a Canon Macro Lens (Canon, Inc., Mellville, NY) on a tripod.

### 3D print geometric fidelity

To assess the geometric fidelity of 3D prints (twisted vase, water drop vase, toe, ear) made using different materials (Epoxy, PDMS, PLA), two types of analyses were performed. Both analyses were based on CAD models obtained after scanning each of the 3D prints using the FaroArm 3D scanner (FARO Technologies, Inc., Lake Mary, FL). The first involved computing the surface area of the CAD models using the Geomagic Wrap 3D imaging software (3D Systems, Inc., Rock Hill, SC). The surface area assessment was based on 36 digital models obtained from 3D scans of the prints (four print geometries and three print materials, each 3D printed three times to have repeats). A statistical analysis was performed across the materials for a given geometry as described in the *Statistical analysis* section below. For the second analysis, the Geomagic software was used to determine the surface deviation between the CAD models generated from 3D scans and the original digital model. This analysis was done for one of PLA, epoxy, and PDMS models in two steps. In a first step, the best fit alignment feature was used to overlay the two CAD models using the high precision fitting option (sample size: 300). In the second step, a 3D color coded mapping of the differences between the original digital model and the CADs from 3D scans was generated through the deviation analysis in millimeters.

### Statistical analysis

For all analysis, the data was found to be normally distributed with α = 0.05 as per a W/S normality test. A one-way analysis of variance (ANOVA) was performed with α = 0.05 to test for the statistical significance of the surface area of 3D prints (twisted vase, water drop vase, toe, ear) across different materials (Epoxy, PDMS, PLA). The sample size for each print geometry was three (n = 3). The null hypothesis was that the surface area of a given 3D print geometry was the same across all three materials. A subsequent post-hoc test, the Tukey-Kramer procedure, was performed upon rejection of the null hypothesis to distinguish the statistical significance among material sets. Similarly, for the mechanical tests, a one-way ANOVA was performed at α = 0.05 to determine the statistical significance of the elastic moduli of PDMS (Sylgard 184) samples made using different processing techniques (cast [17], 3D printed using previously reported settings [6], and 3D printed using the EGO optimized settings). The null hypothesis was that the processing technique will have no significant effect on elastic modulus of PDMS at α = 0.05. A subsequent post-hoc test, the Bonferroni corrected procedure given the sample sizes were different, was performed upon rejection of the null hypothesis to distinguish the statistical significance between the 3D print processing methods.

## Supporting information

S1 Fig Examples of cylinder and cube scoring using the rubrics. Cylinder and cube scores for print characteristics (response variables) that include fusion, infill, and stringiness for the cylinder, and fusion and bottom for the cube. For each response variable, an example of a low (0/10), middle (5/10), and high (10/10) score print is shown. The cylinder runs were sliced using the Replicator G (Skeinforge) CAD slicing software while the cube was sliced using the Simplify 3D slicer. Scale bars are 5 mm.
(PDF)

S2 Fig Stereomicroscope images of 3D printed silicone cylinders. An example of a low (run #28: 19/30), medium (run #44: 26/30), and high (run #52: 30/30) score prints from the 1st generation, 3rd generation, and 4th generation hill climbs, respectively. Scale bars are 5 mm.
(TIF)

S3 Fig Summary of EGO iterations applied to elastomeric cylinder and cube 3D prints. Using the EGO approach involved a total of 67 cylinder prints to reach a full score optimum using the Replicator G (Skeinforge) slicer. The EGO approach applied to the cube resulted in 167 prints including changes to the CAD slicing program (Replicator G and Simplify 3D).
(TIF)

S1 Table Cylinder score rubrics for layer fusion response variable. Adhesion between printed layers.
(PDF)

S2 Table Cylinder score rubrics for infill response variable. The preservation of hollow space in the center of the cylinder, rather than bulging in of the material into this space.
(PDF)

S3 Table Cylinder score rubrics for stringiness response variable. Adhesion of the first layers that often overhang like strings despite layer fusion in the bulk print.
(PDF)

S4 Table Print parameter space factors (Skeinforge slicer). Range and selected levels of print parameters from the open source Skeinforge slicer (Replicator G software).
(PDF)

S5 Table Physical parameter space factors. Selections from the physical parameter space.
(PDF)

S6 Table Cube score rubrics for layer fusion response variable. Adhesion between printed layers.
(PDF)

S7 Table Cube score rubrics for bottom response variable. Integrity of the cube base.
(PDF)

S8 Table Print parameter space factors (Simplify 3D slicer). Range and selected levels of print parameters from the commercially sold Simplify 3D slicer.
(PDF)
