# Supplementary material for: Expert-guided optimization for 3D printing of soft and liquid materials
Source: PLoS One. 2018 Apr 5;13(4):e0194890. doi: 10.1371/journal.pone.0194890 (PMC5886457; doi:10.1371/journal.pone.0194890)
Supplement: S1 Fig — Cylinder and cube scores for print characteristics (response variables) that include fusion, infill, and stringiness for the cylinder, and fusion and bottom for the cube. For each response variable, an example of a low (0/10), middle (5/10), and high (10/10) score print is shown. The cylinder runs were sliced using the Replicator G (Skeinforge) CAD slicing software while the cube was sliced using the Simplify 3D slicer. Scale bars are 5 mm. (PDF) [file pone.0194890.s001.pdf]

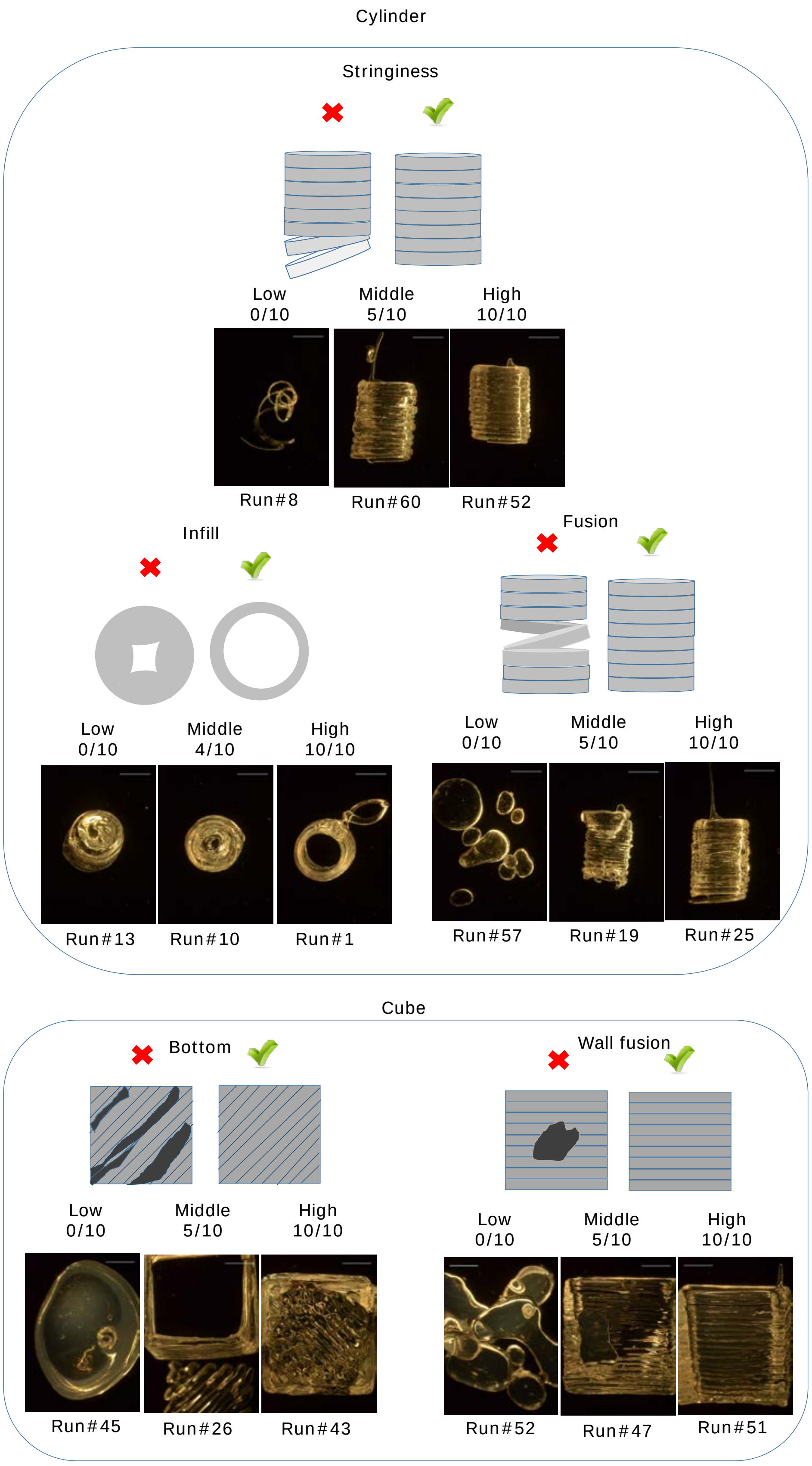
Cylinder
Stringiness
Low
0/10
Middle
5/10
High
10/10
Run#8
Run#60
Run#52
Infill
Low
0/10
Middle
4/10
High
10/10
Run#13
Run#10
Run#1
Fusion
Low
0/10
Middle
5/10
High
10/10
Run#57
Run#19
Run#25
Cube
Bottom
Low
0/10
Middle
5/10
High
10/10
Run#45
Run#26
Run#43
Wall fusion
Low
0/10
Middle
5/10
High
10/10
Run#52
Run#47
Run#51
