# Supplementary figures and images for: Expert-guided optimization for 3D printing of soft and liquid materials
Source: PLoS One. 2018 Apr 5;13(4):e0194890. doi: 10.1371/journal.pone.0194890 (PMC5886457; doi:10.1371/journal.pone.0194890)

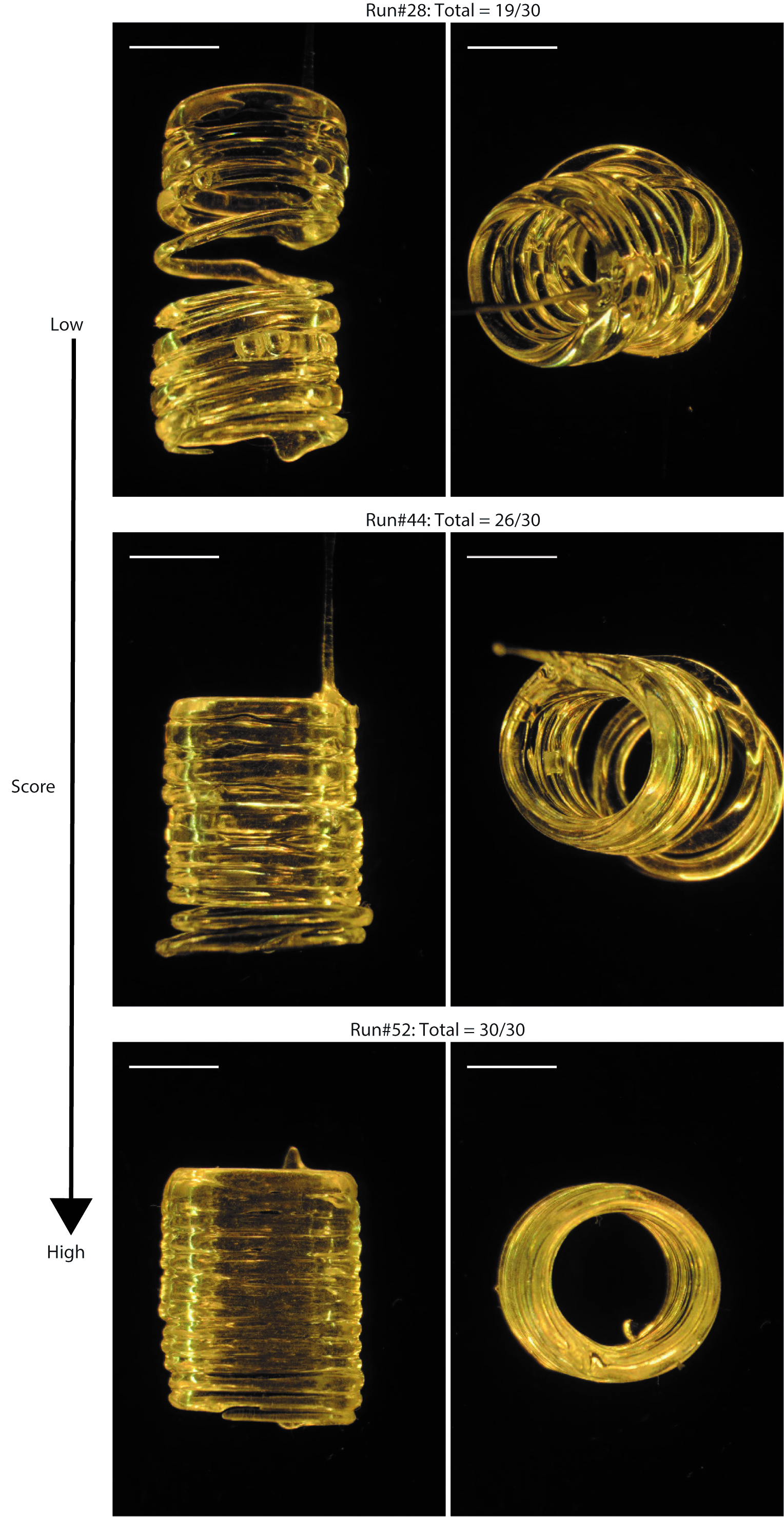

Supplement: S2 Fig — An example of a low (run #28: 19/30), medium (run #44: 26/30), and high (run #52: 30/30) score prints from the 1st generation, 3rd generation, and 4th generation hill climbs, respectively. Scale bars are 5 mm. (TIF) [file pone.0194890.s002.tif]

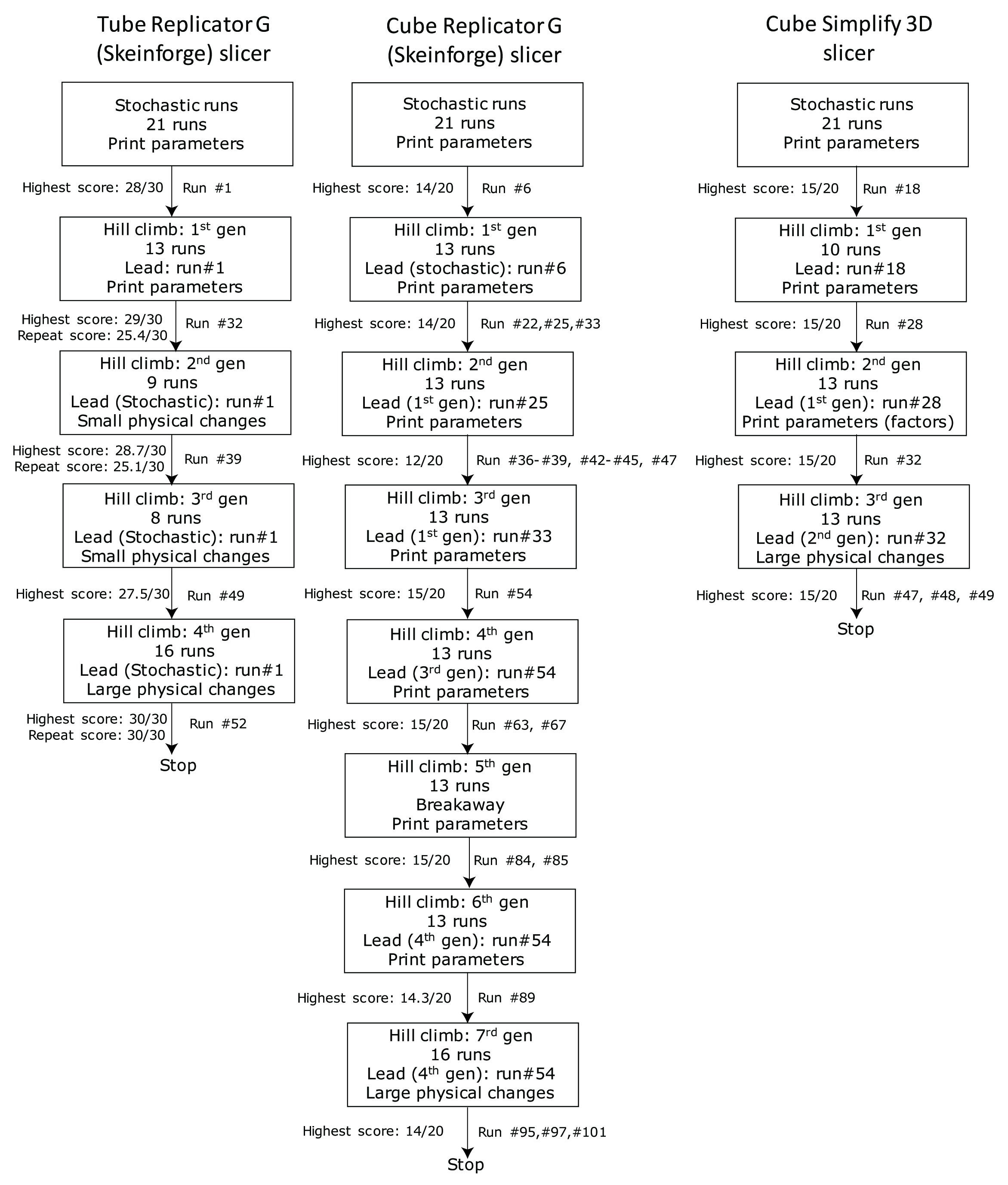

Supplement: S3 Fig — Using the EGO approach involved a total of 67 cylinder prints to reach a full score optimum using the Replicator G (Skeinforge) slicer. The EGO approach applied to the cube resulted in 167 prints including changes to the CAD slicing program (Replicator G and Simplify 3D). (TIF) [file pone.0194890.s003.tif]
