# Supplementary material for: Expert-guided optimization for 3D printing of soft and liquid materials
Source: PLoS One. 2018 Apr 5;13(4):e0194890. doi: 10.1371/journal.pone.0194890 (PMC5886457; doi:10.1371/journal.pone.0194890)
Supplement: S1 Table — Adhesion between printed layers. (PDF) [file pone.0194890.s004.pdf]

Cylinder fusion	
**Description**	A cylinder is fused when the layers are joined together, without spaces or tears in between.<br>Please choose one of the following to describe the extent of cylinder fusion for the following cylinder shown (No layer fusion = 0; Fully fused layers = 10):
**Score**	**Rubric**
0	No apparent cylinder structure, mostly disconnected pieces of string/filaments.
1	No apparent cylinder structure, one long piece of string (the cylinder turns and twists can be seen throughout the string)
2	No cylinder structure, a few (2 to 3) layers fused together.
3	No cylinder structure, some (4 to 6) layers fused together.
4	A cylinder shape, but with major damage (e.g., ripped wall). A cylinder skeleton.
5	A cylinder shape with one fully fused wall side, but other walls sides are partially fused.
6	A cylinder shape with no major rips or cuts, but poor joining between many layers.
7	A cylinder shape with two completely fused structures with a major cut between them.
8	A cylinder shape where some lack of fusion of layers (not all on one wall side) is only apparent after physical inspection.
9	A cylinder shape that looks fully fused, after physical inspection (as seen from tweezer pressures) minor unfused layer can be seen.
10	A cylinder shape with all layers fused. Physical stretching (as seen from tweezer pressures) does not tear the cylinder.
