# Supplementary material for: Expert-guided optimization for 3D printing of soft and liquid materials
Source: PLoS One. 2018 Apr 5;13(4):e0194890. doi: 10.1371/journal.pone.0194890 (PMC5886457; doi:10.1371/journal.pone.0194890)
Supplement: S2 Table — The preservation of hollow space in the center of the cylinder, rather than bulging in of the material into this space. (PDF) [file pone.0194890.s005.pdf]

Cylinder infill	
**Description**	A cylinder has no infill if it is hollow.<br>Please choose one of the following to describe the extent of cylinder infill for the following cylinder shown (Full = 0, Hollow = 10):
**Score**	**Rubric**
0	Cylinder is full from beginning to end or cylinder shape cannot be distinguished.
1	Cylinder is ~75% (3/4) filled.
2	Cylinder is ~50%-60% filled.
3	Cylinder is filled at some part of the ends about ~30%.
4	Cylinder has a severe hourglass: the top and bottom are hollow, but the middle part has full infill.
5	Cylinder has a mild hourglass: the top and bottom are hollow, but the middle part has some thin infill.
6	Cylinder does not seem to have an hourglass shape, but some parts slightly bulge in although doesn’t block the view of the other end.
7	Cylinder is hollow but the wall thickness varies dramatically.
8	Cylinder is hollow but the wall thickness varies mildly.
9	Cylinder is almost perfectly hollow, walls are uniform but too thick.
10	The cylinder is perfectly hollow.
