# Supplementary material for: Expert-guided optimization for 3D printing of soft and liquid materials
Source: PLoS One. 2018 Apr 5;13(4):e0194890. doi: 10.1371/journal.pone.0194890 (PMC5886457; doi:10.1371/journal.pone.0194890)
Supplement: S3 Table — Adhesion of the first layers that often overhang like strings despite layer fusion in the bulk print. (PDF) [file pone.0194890.s006.pdf]

Cylinder end stinginess	
**Description**	A cylinder end has stringiness when the filament at the end of the cylinder hangs loose or came off and is not fused.<br>Please choose one of the following to describe the extent of cylinder end stringiness for the cylinder shown (Ends are stringy = 0, No end stringiness = 10):
**Score**	**Rubric**
0	No apparent cylinder structure.
1	Cylinder is one long string that twists into a cylinder-looking shape or a cylinder skeleton.
2	Cylinder has only 1-4 fused layers, but is mostly strings that twists into a cylinder-looking shape.
3	A cylinder shape, but middle of cylinder is stringy (several mid-layers not fused).
4	Well-defined cylinder structure with 2-4 layers of one or both end strings came off.
5	Well-defined cylinder structure but last layer of one or both ends came off.
6	Well-defined cylinder structure with 2-4 layers of hanging string (unfused) at one end.
7	Well-defined cylinder structure but last layer on one end is hanging/unfused.
8	Well-defined cylinder structure, the end seems fused, but pressure (tweezer squeeze) shows the last layer**s** are only semi-attached to the cylinder.
9	Well-defined cylinder structure, the end seems fused, but pressure (tweezer squeeze) shows the last layer to be nearly completely attached to the cylinder.
10	Well-defined cylinder structure with no hanging strings. Pulling the top and bottom layers does not detach the layers.
