# Supplementary material for: Expert-guided optimization for 3D printing of soft and liquid materials
Source: PLoS One. 2018 Apr 5;13(4):e0194890. doi: 10.1371/journal.pone.0194890 (PMC5886457; doi:10.1371/journal.pone.0194890)
Supplement: S4 Table — Range and selected levels of print parameters from the open source Skeinforge slicer (Replicator G software). (PDF) [file pone.0194890.s007.pdf]

Factors on the print parameter space (Skeinforge slicer)			
Factors	Factor definition	Factor range	Selected factor levels
Speed (mm/s)	Sets the feed rate and flow rate.	2 to 50	7, 10, 13, 17, 19, 20, 23, 25, 26
Filament packing density	Effective filament diameter after accounting for the pinch wheel teeth grab on the filament.	0.7 to 1	0.2, 0.4, 0.5, 0.52, 0.54, 0.56, 0.58, 0.6, 0.75, 0.8
Layer height (mm)	Height of the layers the object is cut into, in the z direction.	0.1 to 1	0.28, 0.381, 0.4064, 0.6, 0.635, 0.84, 0.9652, 1.2, 1.2192, 1.346, 1.52, 2.1082 (based on nozzle diameter)
Tower	Extrudes a disconnected region for a few layers, then go to another disconnected region and extrude there.	On/Off	On/Off
Comb	Bends the extruder travel paths around holes in the slices, to avoid stringers.	On/Off	On/Off
Retraction distance (mm)	Amount the extruder retracts the extruded filament whenever an extruder stop is commanded.	0 to 100	0.1, 0.2, 0.3, 0.4, 0.5, 0.6, 1, 2
Solid surface thickness (mm)	Number of solid layers that are at the bottom, top, plateaus and overhang.	0 to 5	1, 2, 3, 4, 5, 6
Infill pattern	Pattern used to create infill	grid circular, grid hexagonal, grid rectangular, line	grid circular, grid hexagonal, grid rectangular, line
Grid extra overlap (ratio)	Amount of extra overlap added when extruding the grid to compensate for the fact that when the first thread going through a grid point is extruded, since there is nothing there yet for it to connect to it will shrink extra.	0 to 0.5	0, 0.5, 1
Infill solidity (ratio)	Solidity of the infill	0.04 to 0.3	0, 0.5, 1
