# Supplementary material for: Expert-guided optimization for 3D printing of soft and liquid materials
Source: PLoS One. 2018 Apr 5;13(4):e0194890. doi: 10.1371/journal.pone.0194890 (PMC5886457; doi:10.1371/journal.pone.0194890)
Supplement: S5 Table — Selections from the physical parameter space. (PDF) [file pone.0194890.s008.pdf]

Factors on the physical parameter space			
Factors	Factor definition	Factor range	Selected factor levels
Bath type	Polyacrylic bath used to support 3D prints	In this study, restricted to Carbomers	Carbopol 940, Carbopol 1342, Carbopol 941, Carbopol Ultrez 10, Carbopol 934, Carbopol 974, ETD2020, Pemulen 1621
Bath concentration (% w/v)	Concentration of Carbopol 940 and ETD2020 support bath used to support prints	Many possibilities	0.1, 0.2, 0.5, 1, 2
Ink type	Silicone elastomer used as extrusion (ink) material for 3D printing	Many possibilities, the primary focus is to 3D print Sylgard 184	Sylgard 184, Sylgard 186, Sylgard 567, Dow Corning 3-4241
Bath pH	pH of polymeric support bath used to support prints	0 to 14	5, 6.5, 7, 7.5, 8
Ink concentration (Catalyst:Base)	Silicone elastomer catalyst to base ratio used as extrusion (ink) material for 3D printing	Many possibilities, which can be bound by manufacturer recommendations	1:10, 1.1:10, 1.2:10, 1.3:10, 1.4:10, 1.5:10, 1.8:10, 0.5:10, 2:10
Bath stirring time (h)	Period used to stir the polymeric support bath in preparation for 3D printing	Many possibilities	4, 8, 12
Ink curing time (h)	Period the silicone elastomer was left to heat cure in oven	Many possibilities, which can be bound by manufacturer recommendations	3, 7, 18
