# Supplementary material for: Expert-guided optimization for 3D printing of soft and liquid materials
Source: PLoS One. 2018 Apr 5;13(4):e0194890. doi: 10.1371/journal.pone.0194890 (PMC5886457; doi:10.1371/journal.pone.0194890)
Supplement: S6 Table — Adhesion between printed layers. (PDF) [file pone.0194890.s009.pdf]

Cube fusion	
**Description**	A cube wall is fused when the layers are joined together without any tears in between. Please choose one of the following to describe the extent of the cube wall fusion for the cube below (No layer fusion = 0; Fully fused layers = 6):
**Score**	**Rubric**
0	No apparent cube structure.
1	A cube shape, but with major damage; rough cube skeleton distinguishable.
2	A cube shape, one or more walls visibly damaged & intersection between two walls not fused.
3	A cube shape, but one or more walls are visibly damaged.
4	A cube shape, but one or more walls are not fused.
5	A cube shape, walls seem okay, but intersection between two walls not fused.
6	A cube shape with all layers and wall intersections seem fused.

Note: The maximum cube fusion score is 6, which is scaled to 10 for the final score count that is out of 20.
