# Supplementary material for: Expert-guided optimization for 3D printing of soft and liquid materials
Source: PLoS One. 2018 Apr 5;13(4):e0194890. doi: 10.1371/journal.pone.0194890 (PMC5886457; doi:10.1371/journal.pone.0194890)
Supplement: S7 Table — Integrity of the cube base. (PDF) [file pone.0194890.s010.pdf]

Cube bottom	
**Description**	Please choose one of the following to describe the condition of the cube bottom shown from a top and underneath view of the cube:
**Score**	**Rubric**
0	Cube cannot be distinguished, therefore neither can a bottom.
1	Cube is bottomless (walls are there though).
2	Cube is full from beginning to end (and some silicone may be bulging out).
3	Cube bottom is made of a loop pattern of only one layer loosely attached. Area between loops is empty.
4	Cube bottom is made of a loop pattern of only one layer that is attached. Area between loops is empty.
5	Cube has a bottom completely detached from the rest of cube.
6	Cube bottom is made of some inconsistent pattern. Portions of the bottom are filled, some chunks are empty.
7	Cube bottom is made of overlaying loop patterns (or some pattern), some small holes are seen and/or the perimeter of the cube bottom is loosely attached.
8	Cube bottom has no inconsistencies/holes, but the bottom appears to be too thick (thicker than ~ 2 mm).
9	Cube bottom consists of one ~ 2 mm layer that with inspection (pulling/poking) has some lines not well fused and/or one of the bottom perimeter rims is loosely attached.
10	The cube has a nice bottom without holes and without excess filling (~2 mm thickness).
