# Supplementary material for: Expert-guided optimization for 3D printing of soft and liquid materials
Source: PLoS One. 2018 Apr 5;13(4):e0194890. doi: 10.1371/journal.pone.0194890 (PMC5886457; doi:10.1371/journal.pone.0194890)
Supplement: S8 Table — Range and selected levels of print parameters from the commercially sold Simplify 3D slicer. (PDF) [file pone.0194890.s011.pdf]

Factors on the print parameter space (Simplify 3D slicer)			
Factors	Factor definition	Factor range	Selected factor levels
Nozzle diameter (mm)	Diameter of syringe nozzle.	0 to 200	0.6, 0.635, 0.84, 0.9652, 1.2, 1.2192, 1.52, 2.1082
Primary layer height (mm)	Thickness of each printed outline layer.		
Bottom Solid Layers	Number of solid layers to require at the bottom of the part.	0 to 99999	0,1, 2, 3, 4
Default printing speed (mm/min)	Initial speed used for all printing movements.	0.1 to 100000	10 mm/s, 50 mm/s, 70 mm/s, 100 mm/s, 150 mm/s
External Fill Pattern	Determines the infill pattern used for the external surfaces of the part.	Concentric, rectilinear	Concentric, rectilinear
Retraction	Reverses filament direction at the end of a loop to help prevent stringing.	On/Off	On/Off
Outline/Perimeter Shells	Number of shells to use for the extruder skin of the part.	0 to 99999	1, 2, 3
Single outline corkscrew printing mode	Gradually increment the Z-axis to avoid any layer change seams.	On/Off	On/Off
Start points	Determines location of layer start point for 3D printing.	Random, Optimize, Choose	Random, Optimize
Outline overlap (%)	Percentage of extrusion width that will overlap with outline perimeters.	0 to 99	10, 30, 40
Thin wall behavior	Provides options for processing thin walls.	Use perimeters, gap fill	Use perimeters, gap fill
Ooze control behavior	Provides options for controlling ooze.	Retract when crossing, Force retraction between layers, Minimum travel for retraction, Retract during wipe, Only wipe for outer-perimeter	Retract when crossing, Force retraction between layers, Retract during wipe
Tool change retraction distance (mm)	How much plastic to pull back into nozzle after a tool change.	0 to 200	5, 12
